# A role for reactive oxygen species in the resolution of persistent genomic instability after exposure to radiation

**DOI:** 10.1093/jrr/rrt183

**Published:** 2014-03

**Authors:** Erica Werner, Ramesh Kandimalla, Huichen Wang, Paul W. Doetsch

**Affiliations:** 1 Department of Biochemistry, Emory University School of Medicine, 1510 Clifton Road, Rm 4019, Atlanta, GA 30322, USA; 2 Department of Radiation Oncology, Winship Cancer Institute, Emory University School of Medicine, Atlanta, GA, USA

**Keywords:** reactive oxygen species, genomic instability, senescence

## Abstract

Reactive oxygen species (ROS) are involved in amplifying the radiation-induced damage to macromolecules as well as in downstream early responses leading to DNA repair or cell death and in delayed responses leading to tissue damage and/or tumorigenesis. We found in immortalized normal human bronchial epithelial cells (HBEC-3KT) following exposure to 1 Gy low (X-rays) or high (Fe ions) LET radiation that ROS levels persist elevated for up to eight population doublings (2 weeks) in the progeny of cells that repaired the radiation-induced damage and survived. To evaluate the function of persistently elevated ROS levels, we interrogated cells over the same period for genomic instability, proliferation and senescence biomarkers and evaluated whether ROS are functionally related to any of these phenotypes. We found that increased ROS production overlapped temporally with the persistence of reporters for genomic instability, increased micronucleus frequency and the presence of γH2AX-53BP1 positive ionizing radiation-induced foci. Although low LET radiation at high doses and high LET radiation induced a senescence-like phenotype dependent on ATM and p38 MAPK activity, this pathway was not a causative factor in ROS generation and did not affect cell proliferation rates at day 7 post-irradiation. On the contrary, when this pathway was inhibited, ROS levels increased further and micronucleus formation frequency was reduced. Because these results suggest a positive effect for increased ROS levels in the resolution of persistent micronucleus formation, their role was further confirmed by exposure to exogenous hydrogen peroxide, which resulted in a reduced micronucleus frequency. These results implicate ROS as an effector in the resolution of genomic instability and suggest that the senescence phenotype induced by high LET radiation or high low LET radiation dose promotes genomic instability by interfering with the cellular mechanism regulating ROS levels or the DNA repair machinery.

